# Potential data for feasibility assessment and deployment of a 6.7 MW floating solar PV plant in Hatirjheel Lake, Dhaka, Bangladesh

**DOI:** 10.1016/j.dib.2024.110586

**Published:** 2024-06-06

**Authors:** Md. Imamul Islam, Ahmed Al Mansur, Mohd Shawal Jadin, D.M. Saaduzzaman, Md. Naiem-Ur-Rahman

**Affiliations:** a Faculty of Electrical and Electronics Engineering Technology, Universiti Malaysia Pahang Al-Sultan Abdullah, 26600 Pekan, Pahang, Malaysia; b Department of Electrical and Electronic Engineering, Green University of Bangladesh, Purbachal American City, Kanchon 1460, Rupganj, Narayanganj, Bangladesh

**Keywords:** Floating solar PV, Feasibility assessment, Hatirjheel Lake, Climate, Potential data

## Abstract

Floating solar photovoltaic has emerged as a highly sustainable and environmentally friendly solution worldwide from the various clean energy generation technologies. However, the installation of floating solar differs from rooftop or ground-mounted solar due to the significant consideration of the availability of water bodies and suitable climatic conditions. Therefore, conducting a feasibility analysis of the suitable climate is essential for installing a floating solar plant on water bodies. These data are evaluated for the viability of installing a 6.7 MW floating solar power plant on Hatirjheel Lake in Dhaka, Bangladesh. The feasibility analysis incorporated various climatic data, such as temperature, humidity, rainfall, sunshine hours, solar radiation, and windspeed, obtained from Meteonorm 8.1 software and the archive of the Bangladesh Meteorological Department. Besides, this study gathered and analyzed the energy demands of the local grid substation operated by Dhaka Power Distribution Company, to determine the appropriate capacity and architecture of the power plant. The power plant design was conducted using the PVsyst 7.3 software, which determined the necessary equipment quantities, DC energy generation capacity, and the energy injected into the grid in MWh. The study also calculated the Levelized Cost of Energy per kilowatt-hour and the payback period for the system, which indicates the economic viability of installing the system. Furthermore, the acquired dataset possesses significant potential and can be utilized for the establishment of all sorts of solar power plants, including floating solar plants, in any location or body of water within the Dhaka Metropolitan area.

Specifications Table

**Table:** 

Subject	Renewable Energy, Sustainability, and the Environment
Specific subject area	Feasibility analysis of a 6.7 MW floating solar PV power plant in a lake of Dhaka Metropolitan area
Data format	Raw, Simulated, Filtered
Type of data	Table
Data collection	The procedure for gathering data incorporated a wide variety of sources and devices. The meteorological information about Hatirjheel Lake in Dhaka, encompassing temperature, precipitation, humidity, hours of sunshine, and solar radiation, wind speed was acquired from Mateonorm 8.1 and the repository of the Bangladesh Meteorological Department (BMD). To fulfill a portion of the energy demand of the adjacent grid substation, pertinent data was obtained from Dhaka Power Distribution Company (DPDC). The simulated data of the 6.7 MW Floating Photovoltaic plant was generated and collected using PVsyst 7.3, a commonly used application for evaluating solar systems.
Data source location	Institution: Green University of Bangladesh City/Town/Region: Hatirjheel Lake, Dhaka North City Corporation, Dhaka Country: Bangladesh Latitude and longitude (and GPS coordinates, if possible) for collected samples/data: 23.7496 and 90.3968 (23◦ 44′ 58.47′′ N, 90◦ 23′ 48.35′′ W)
Data accessibility	Repository name: Mendeley Data Data identification number: 10.17632/hpdjdhpbgf.1 Direct URL to data: https://data.mendeley.com/datasets/hpdjdhpbgf/1
Related research article	M. I. Islam et al., “Feasibility analysis of floating photovoltaic power plant in Bangladesh: A case study in Hatirjheel Lake, Dhaka,” Sustain. Energy Technol. Assessments, vol. 55, no. September 2022, p. 102,994, 2023, doi:10.1016/j.seta.2022.102994. [1]

## 1 Value of the Data

• The existing data sets are useful to develop and improve the effectiveness of ground-mounted or rooftop solar and floating solar PV facilities in Dhaka city, as well as to analyze the potential for energy production using solar PV, particularly for the floating solar power plant in Hatirjheel lake and the others accessible water bodies in Dhaka.
• A large range of stakeholders notably the academicians, developers and engineers, policymakers, and public officials, as well as the energy firms could benefit from this data.
• These data might be utilized for periodical analysis to determine solar power generation, and machine learning-based prediction model development, and to measure the risk and opportunity to develop RE installation in the region.
• Further research could be conducted using these data to examine the efficacy of floating solar PV systems and solar PV systems installed on rooftops in the city of Dhaka.

## 2 Background

The imperative need to assess the feasibility and potential installation of a 6.7 MW floating solar PV plant in Hatirjheel Lake, Dhaka, Bangladesh, is the impetus for collecting this dataset. Motivating this undertaking is the need to meet the growing demand for sustainable energy solutions, in addition to the unique capabilities that floating solar technology offers [2]. An exhaustive evaluation of meteorological variables (including temperature, precipitation, humidity, and solar radiation), fluctuations of wind speed, usage of energy, and a complete simulation of the FPV facility are duly considered. The base of this dataset is the environmentally friendly energy integration idea, which emphasizes the significance of concrete proof in the process of making choices [3]. The dataset facilitates thorough implementation planning and viability studies by providing comprehensive details on the energy and environmental landscape. The objective is to assist policymakers, academicians, and stakeholders in their understanding of sustainable solar project design and thereby contribute to the body of knowledge on renewable energy.

## 3 Data Description

The present dataset, located on the Mendeley Data Repository [4], refers to the assessment and execution of a 6.7 MW floating photovoltaic solar energy facility situated in Hatirjheel Lake, Dhaka, Bangladesh. The dataset comprises a variety of simulated, 11-year real-time climatic data (from 1st January 2013 to 31st December 2023) and specific technical and economic data that may contribute to the evaluation of the viability of deploying floating solar power in any body of water in Dhaka to fulfill sustainable development goal 7 [5]. The dataset is organized into several segments in the Mendeley data. The dataset named “Potential Data” includes the simulated data from Meteonorm 8.1, PVsyst 7.3, and the real-time energy demand and real-time daily wind speed for two years. However, the daily wind speed and direction in Dhaka for the years 2020 and 2021 have been demonstrated in this dataset because wind speed is one of the vital climatic factors that need to be considered before designing and implementing a floating solar plant. On the other hand, the dataset titled "11 Years Climatic Data from BMD and Other Specifications" in Mendeley Data contains the monthly real-time climates of Dhaka over 11 years (from 2013 to 2023). It also includes technical specifications, economic analysis, the levelized cost of Energy (LCOE), and the payback period. The meteorological data provided by BMD in real-time comprises monthly measurements of the maximum, minimum, and average temperature, monthly duration of sunlight, monthly precipitation, monthly levels of humidity, and monthly wind speed. This meteorological data is essential for conducting a feasibility study of the FPV plant.

### 3.1 Meteonorm 8.1 data

**Fig. 1:**
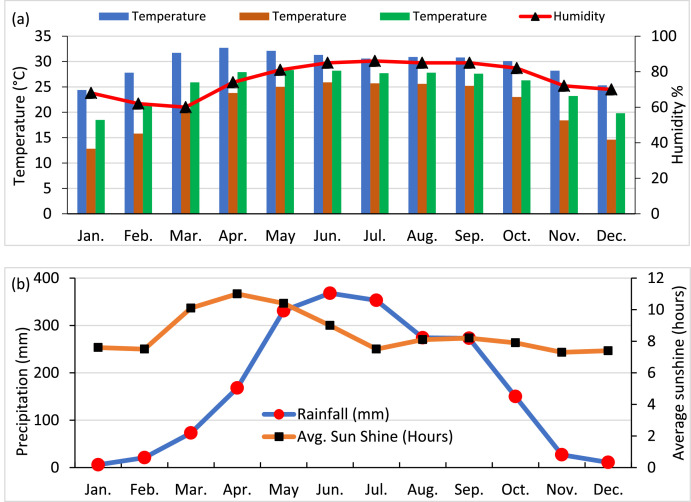
Climatic data of Hatirjheel Lake obtained from Meteonorm 8.1: (a) Monthly max, min, and average temperature, and average humidity; (b) Monthly average rainfall, and sunshine hours.

**Fig. 2:**
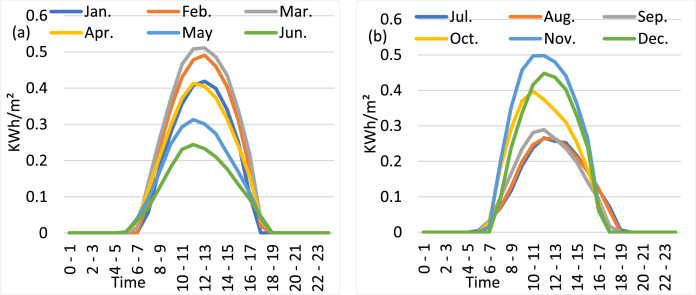
Monthly DNI in KWh/m²: (a) From January to June, (b) From July to December.

The data from Meteonorm 8.1 includes the “Climate of Hatirjheel, Dhaka (monthly maximum, minimum, and average temperature in degrees Celsius, rainfall measurements in millimeters, average sunshine hours, and relative humidity levels in percentage), and the average hourly direct normal solar irradiation (DNI) in KWh/m² by month”. Fig. 1 displays the monthly average climatic data of Hatirjheel Lake obtained from Meteonorm 8.1. On the other hand, Fig. 2 displays the monthly direct normal solar irradiation (DNI) in KWh/m².

### 3.2 PVsyst simulation data

**Fig. 3:**
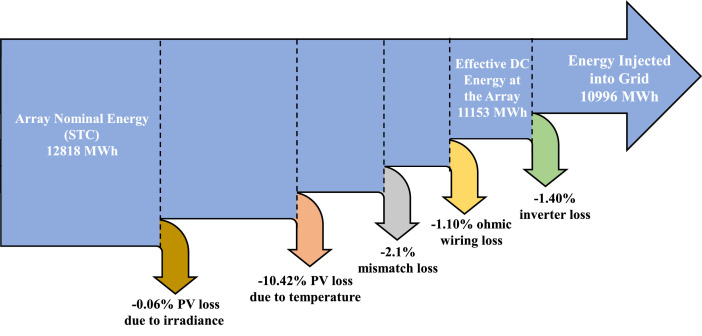
Various losses in the FPV system from PVsyst simulation.

The PVsyst simulation data includes the normalized production, performance ratio (PR), and the main simulated results of the 6.7 MW FPV plant. The monthly average of useful energy production, system losses, PR, effective energy at the end of the array, and energy injected into the grid in MWh have been displayed there from the PVsyst simulation, correspondingly. The simulation results of the 6.7 MW FPV plant from the PVsyst are shown in Table 1. The results describe the average monthly array yield, system loss, system yield, effective energy generation, supplied energy to the grid, and performance ratio. In the PVsyst simulation, the plant has the annual array nominal energy at STC 12,818 MWh, however, due to the several losses in the array system including solar irradiance loss, temperature, mismatch, and ohmic wiring loss, the annual effective DC energy at the end of the array is 11,153 MWh. After considering the inverter loss, about 10,996 MWh of energy will be injected into the grid. The loss diagram of the simulation study is shown in Fig. 3. As the system is directly grid-connected and there is no storage system, hence the loss due to battery storage has been ignored.

**Table 1:** PVsyst simulation result.

Items	Jan.	Feb.	Mar.	Apr.	May	Jun.	Jul.	Aug.	Sep.	Oct.	Nov.	Dec.
Array Yield (kWh/kWp/day)	4.24	5	5.18	4.96	4.7	3.96	4.15	4.14	4.24	4.55	4.81	4.32
System Loss (kWh/kWp/day)	0.061	0.068	0.07	0.07	0.067	0.058	0.06	0.061	0.06	0.064	0.065	0.059
System Yield (kWh/kWp/day)	4.18	4.93	4.89	4.89	4.63	3.9	4.09	4.08	4.18	4.49	4.75	4.26
Effective energy at array output (MWh)	889	946	1086	1006	985	804	871	869	861	954	976	906
Energy injected into the grid (MWh)	876	933	1071	992	971	992	858	856	849	941	963	894
Performance Ratio (PR)	0.867	0.84	0.814	0.828	0.839	0.855	0.854	0.852	0.847	0.847	0.86	0.871

### 3.3 Grid substation demand data

The related research article [1], specifically concentrated on fulfilling a certain fraction of the energy demands for the Moghbazar substation, which is the closest grid substation to Hatirjheel Lake. The monthly energy demand of the various feeders in this substation was obtained from the DPDC and is documented in the Energy Demand sheet of the Excel file. However, before designing the floating structure and selecting the necessary equipment for the plant, it was crucial to determine the energy demand of the closest grid substation. This was vital since the plant had the potential to meet a certain portion of the energy demands of the local area. The monthly energy consumption of the Moghbazar grid substation was taken from the yearly reports of DPDC for the following feeders: Ambagan, Desh TV, Doctor's Lane, Ispahani, Modhubag, Nayatola, Padma, and Sonalibag. The system was intended to meet 12.5 % of the annual local energy consumption, which amounts to 87.431 MKWh. Feeder-wise total yearly energy demand and monthly total energy demand are displayed in Table 2, Table 3 respectively.

**Table 2:** Feeder-wise yearly energy demand (MKWh) of Moghbazar 132/33/11 kV grid substation under DPDC.

Ambagan	Desh TV	Doctorʼs Lane	Ispahani	Modhubag	Nayatola	Padma	Sonalibag
13.172144	10.11376	7.648295	11.63916	10.08226	16.99989	9.388267	8.387606

**Table 3:** Monthly energy demand (MKWh) of Moghbazar 132/33/11 kV grid substation under DPDC.

Jan.	Feb.	Mar.	Apr.	May	Jun.	Jul.	Aug.	Sep.	Oct.	Nov.	Dec.
4.747	4.783	7.604	8.607	8.398	7.957	8.160	8.584	8.826	8.567	6.012	5.187

### 3.4 Real-time BMD data

**Fig. 4:**
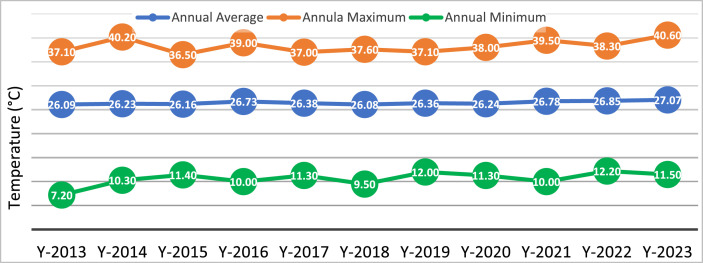
Annual maximum, minimum, and average temperature of Dhaka from the year 2013 to 2023.

The real-time climate and conditions are crucial in determining the design, feasibility, and performance analysis of the FPV plant. The meteorological data of Dhaka from 2013 to 2023 has been obtained from BMD to get insight into the local environment and assist with feasibility analysis and decision-making. Temperature changes, including maximum, minimum, and average values, are crucial considerations in the planning and operation of FPV plants, among other environmental conditions. The fluctuation of these changes directly affects the effectiveness of solar panels and the total energy production of the system. Comprehending and considering these temperature trends is crucial for enhancing the cooling systems, capacity, and overall efficiency of the FPV plant to guarantee optimal energy production all year round. Fig. 4 illustrates the yearly maximum, minimum, and average temperature data for Dhaka acquired from BMD for the last decade. The average temperature of Dhaka has consistently remained close to room temperature, which did not exceed more than 27.07 °C over the last decade. However, there has been considerable worry over the maximum temperature in the previous two years. Nevertheless, the comprehensive temperature record indicates the appropriateness of establishing the FPV plant in Dhaka.

**Fig. 5:**
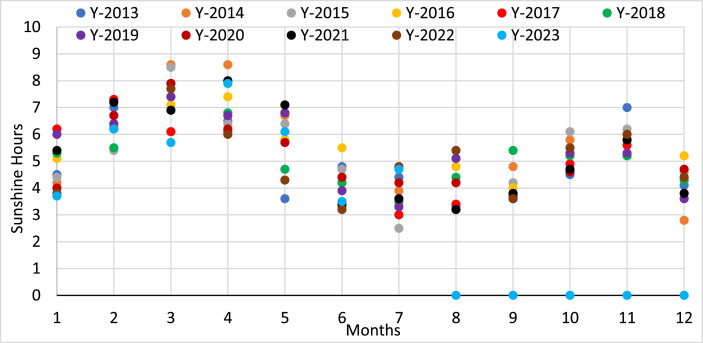
Monthly (2013–2023) sunshine hours data obtained from BMD.

The monthly sunshine hours data from 2013 to 2023 shows that Dhaka has a varying but steady quantity of sunlight throughout the year. March and April often see the highest average sun duration, with a notable decrease from June to September during the monsoon season. With very little fluctuations from year to year, the yearly average sunlight hours are comparatively constant at around 5 h. Notably, the last four months of 2023 show no reported sunlight hours at all, which raises the possibility of a problem with data collection by BMD. Fig. 5 represents the monthly sunshine hours of Dhaka from the year 2013 to 2023.

**Fig. 6:**
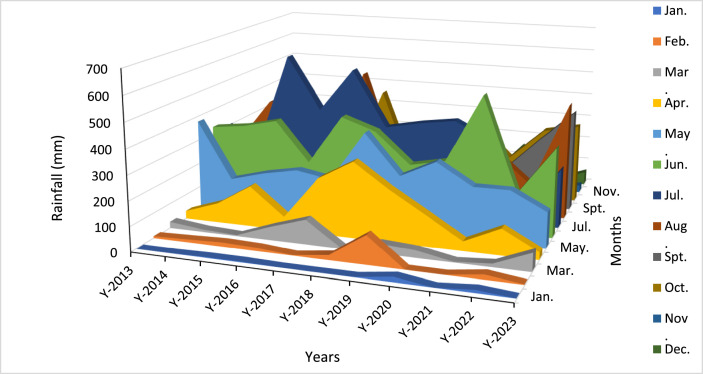
Monthly rainfall in millimeters in Dhaka from 2013 to 2023.

However, the rainfall pattern in Dhaka is distinguished by a prominent monsoon season, with the highest level of precipitation occurring from June to September. The annual precipitation shows significant year-to-year variation, with a range of more than 2800 mm in 2017 to just over 1300 mm in 2022. Typically, the first two months of the year see very little rainfall because of the winter season, however, there can be occasional exceptions. The inherent unpredictability of rainfall highlights the criticality of implementing resilient water management solutions in Dhaka. In addition, a significant issue for waterbodies is the reduction in water levels over the winter and spring seasons. Fig. 6 depicts the monthly precipitation in Dhaka spanning from 2013 to 2023.

**Fig. 7:**
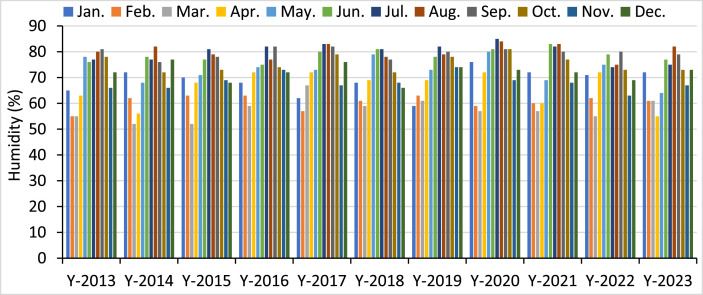
Monthly relative humidity level in Dhaka from 2013 to 2023.

Fig. 7 displays the relative humidity in Dhaka from 2013 to 2023. It shows that Dhaka consistently has high relative humidity throughout the year, with a minor rise during the monsoon season (June-September). The monthly average humidity fluctuates between 70 % and 80 %, suggesting a mostly humid environment. Over 11 years, the average relative humidity stays very constant, with just little variations from year to year. The persistent high humidity in Dhaka highlights the need to choose suitable materials, execute strong maintenance procedures, and optimize operating parameters for FPV plants. This is crucial to guarantee their long-term effectiveness and longevity.

**Fig. 8:**
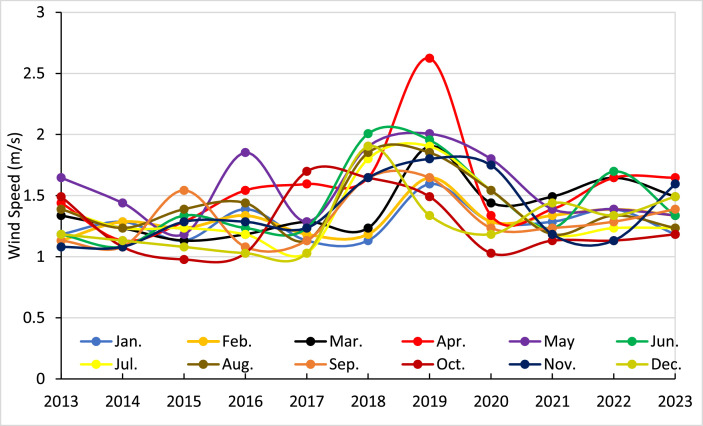
Monthly windspeed of Dhaka from the year 2013 to 2023.

The wind speed plays an essential function in the design of floating solar plants since it directly affects the strength and stability of the system. Strong winds may apply substantial stresses to the floating platform and solar panels, requiring strong anchoring and mooring mechanisms. Moreover, energy production is affected by wind speed since an excessive amount of wind may lead to shadowing and decrease efficiency. Comprehending wind patterns is crucial for maximizing panel arrangement and guaranteeing the secure and effective functioning of the plant. Therefore, the monthly wind speed data collected in real-time during the last 11 years in Dhaka has been regarded as a very significant metric. In addition, to get a comprehensive understanding of the influence of wind speed, the daily wind speed data for the last two years has also been acquired from BMD. Fig. 8 displays the monthly wind speed data spanning from 2013 to 2023, as well as the daily wind speed data specifically for the years 2020 and 2021. The monthly wind seeds dataset indicates that Dhaka consistently receives low wind speeds throughout the year, with average monthly values often falling between 1.0 m/s and 1.8 m/s. Although there are sporadic instances when the speed exceeds 2.0 m/s, these occurrences are rare and usually happen in the months leading up to the monsoon season and during the monsoon season itself (April-September). There are slight variations in the yearly average wind speed from year to year, but there are no notable upward or downward trends across the 11 years. The data indicates that Dhaka has a consistently steady wind pattern, characterized by consistently low wind speeds during most of the year.

## 4 Experimental Design, Materials and Methods

**Fig. 9:**
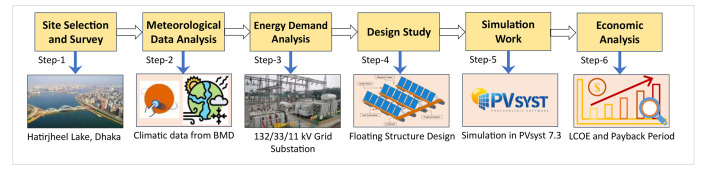
Step-by-step data collection procedure.

This study has adhered to a meticulous procedure to gather all the essential data. Fig. 9 illustrates the sequential procedure used for data collecting in this investigation. To gather the data, the first consideration was selecting an appropriate place for the installation of the FPV. Upon selecting the location, the essential climatic data was obtained using Meteonorm 8.1 software, as well as from the Bangladesh Meteorological Department (BMD) with particular emphasis on the monthly temperature, solar radiation, precipitation, relative humidity, wind speed, and sunlight hours. The data was purchased from the BMD due to the significance of climatic conditions to the construction of an FPV plant. Nevertheless, the climatic data and wind speed indicated the appropriateness of the site. However, before designing the FPV system, the meteorological and grid substation demand data was analyzed properly, and based on the analyzed data, the FPV system was designed. Besides, the LCOE and the Payback period also has been calculated based on the economic analysis.

The Hatirjheel Lake in Dhaka City offers a distinctive prospect for the establishment of an FPV facility, and the site has been chosen based on the physical survey. The extensive expanse of its surface allows for the installation of a huge solar array, reducing the need for acquiring land and possible clashes with agricultural or urban growth. Furthermore, the proximity of the lake to the city center enables seamless integration with the current power system, resulting in fewer transmission losses and related expenses. Although the location presents difficulties such as variations in water levels throughout the year and possible negative effects on the environment, meticulous planning, and efforts to minimize these issues will guarantee the effective establishment of a renewable and efficient FPV system. According to the various literature, the site-specific information is given in Table 4 [[6], [7], [8]].

**Table 4:** Site-Specific Information of Hatirjheel Lake.

Description/Parameters	Unit/Values
Lake Area	302 acres
Water level below the surface	5–9 m
Seasonal water level fluctuations	0.5–2 m
Oxygen Level on the Water	5.3 mgL^−1^
Water Quality: pH/ Electrical conductivity (EC)/ Total dissolved solids (TDS) / Total suspended solids (TSS)/ Total alkalinity	6.51–7.05/ 510–600 µS.cm-1/ 450–590 ppm/ 0.0–0.034 mgL^−1^/ 80- 392 mgL^−1^
Probability of seismic	Less (seismic zone 2)
Probability of flood	Over +5.5 PWD

**Fig. 10:**
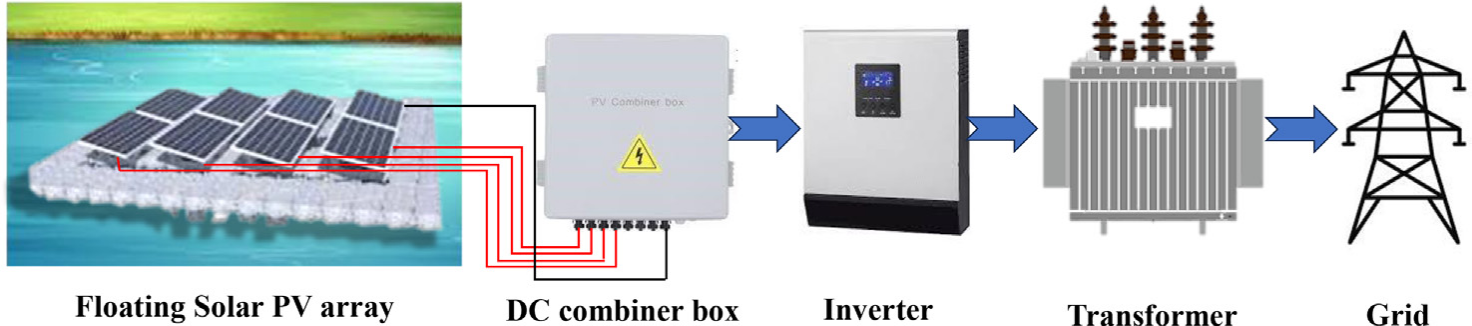
Layout of the floating PV system: Energy generation to transmission.

To design the 6.7 MW FPV plant using PVsyst 7.3 software, several components (PV panels, Inverter, Transformer, etc.) from several renowned manufacturers have been chosen. The software helped to choose and analyze the array size, number of PV panels, number of inverters, and other necessary equipment. Table 4 shows the specifications of the components. The floating PV array is composed of several solar panels that are interconnected in both series and parallel configurations called an array, therefore producing DC power. The DC output from each array is sent into a DC combiner box, where it is consolidated and optimized for transmission. Subsequently, the aggregated DC power is sent to an inverter, which transforms it into AC energy, appropriate for use in the power grid. A transformer is used to further increase the voltage of the AC electricity to meet the specific needs of the electrical grid. Ultimately, the converted AC electricity is sent to the grid connection point, enabling the produced energy to be disseminated to end-users. The layout of the FPV design is shown in Fig. 10 (Table 5).

**Table 5:** Specifications of the used components.

PV panel specification	Unit/Values	Inverter Specification	Unit/Values
Manufacturer	Lungi Solar	Manufacturer	ASEA Brown Boveri
Rated power (W)	350	DC voltage range	525 V- 825 V
Voc (V)	40.40	Vmax DC	1100 V
Isc (A)	11.16	Imax DC	1710 A
Vmp (V)	34.40	Nominal AC power	875 kW
Imp (A)	10.18	Maximum AC power	1050 kW
No. of cells	60×2	Nominal voltage, AC	350 V
Efficiency/module (%)	19.44	Nominal current, AC	1445 A

Economic suitability is one of the major considerations for FPV design and installation. The levelized cost of energy (LCOE) per kWh and the payback period in years are the metrics of economic suitability analysis. The determination of LCOE includes the evaluation of capital expenditures (CAPEX), operating expenditures (OPEX), and the overall generated electricity within the operational timeframe. The LCOE is calculated using the method specified in the following equation [9].

(1) $$LCOE=\frac{\sum_{n=1}^{N}\left(Capex_{n}-Opex_{n}\right)}{\sum_{n=1}^{N}\frac{E_{n}}{{\left(1+r\right)}^{n}}}$$

Here, N is the duration of the evaluation period. Capex_n_ represents the amount of money spent on capital investments in year n. OPEX_n_ refers to the expenses associated with operations in year n. The amount of energy produced in year n is denoted as E_n_. The discount rate (r) quantifies the return that investors want. Table 6 displays the aggregate Capex and Opex over the entire 25-year lifespan of the plant.

**Table 6:** FPV plant LCOE calculation breakdown.

Parameters	Sub-parameters	Unit/Values
Lifetime of the plant, (N)	–	25 years
Total generated energy in 25 years (E_n_)	–	274,900,000 KWh
Discount rate		5.85 %
Total capital expenditures (Capex) in 25 years	Civil works, FPV equipment including PV panels, Inverters, Junction boxes, Consultations, Planning and Design, and Miscellaneous	US$ 14,604,912
Total operational expenditures (Opex) in 25 years	Lake lease, Operation, and maintenances, Salary, Contingency, Tax, Insurance, and Others	US$ 10,662,410

The levelized cost of energy for the FPV plant, calculated using Eq. (1), is expected to be US$ 0.0959 per kWh or BDT 9.015 per kWh (with an exchange rate of BDT 94). According to the National Data Base of Renewable Energy in Bangladesh, the rates per kWh of energy generated by various operational large-scale solar power plants in the country range from US$ 0.110 to US$ 0.171 [1]. The period of payback for the FPV plant at Hatirjheel Lake is determined by multiple factors, including the initial capital expenditures, continuous operational and maintenance expenses, and the money gained from energy sales. The intended FPV plant will inject an annual energy of 10,996,000 kWh into the grid. With the Levelized Cost of Energy of US$ 0.0959/kWh, the FPV plant is expected to save approximately US$ 1054,516.4 per year, as calculated using Eq. (2). Hence, the payback period for this FPV plant is estimated to be around 13.9 years, based on Eq. (3). This is due to the initial investment (total Capex of the system) of US$ 14,604,912 for the entire project. Based on the most recent studies, ground-mounted photovoltaic plants may have a return on investment of 7 to 12 years. The payback time for this FPV system is somewhat longer than 12 years since its cost is greater than that of a ground-mounted PV plant. However, the payback period and LCOE demonstrate the appropriateness and economic feasibility of this FPV technology.

(2) Savingsperyear=Energyinjectedtothegrid(kWh/year)×LCOE

(3) $$Payback\;period=\frac{Total\;Capex}{Savings\;per\;year}$$

## Limitations

Not applicable.

## Ethics Statement

The authors verify that the dataset for Meteonorm 8.1 and PVsyst 7.3 was acquired from licensed software and procured from the BMD. The dataset underwent verification and review, and the corresponding research paper [1] was published by the authors themselves. Besides, the authors have thoroughly reviewed and adhered to the ethical guidelines for publishing in Data in Brief. They affirm that the present study does not include the use of human subjects, animal experimentation, or data obtained from social media sites.

## CRediT authorship contribution statement

**Md. Imamul Islam:** Conceptualization, Investigation, Writing – original draft, Writing – review & editing, Software, Formal analysis, Data curation. **Ahmed Al Mansur:** Conceptualization, Methodology, Writing – original draft, Data curation, Validation, Formal analysis, Investigation. **Mohd Shawal Jadin:** Conceptualization, Writing – review & editing, Data curation, Supervision. **D.M. Saaduzzaman:** Formal analysis, Writing – review & editing, Data curation, Funding acquisition. **Md. Naiem-Ur-Rahman:** Formal analysis, Writing – review & editing, Data curation, Funding acquisition.

## Data Availability

Potential dataset for the viability study of an FPV power plant in Dhaka, Bangladesh (Original data) (Mendeley Data).
